# Using a Web-Based App to Deliver Rehabilitation Strategies to Persons With Chronic Conditions: Development and Usability Study

**DOI:** 10.2196/19519

**Published:** 2021-03-18

**Authors:** Julie Richardson, Lori Letts, Susanne Sinclair, David Chan, Jordan Miller, Catherine Donnelly, Jenna Smith-Turchyn, Sarah Wojkowski, Janelle Gravesande, Adalberto Loyola Sánchez

**Affiliations:** 1 School of Rehabilitation Science Faculty of Health Sciences McMaster University Hamilton, ON Canada; 2 Department of Health Research Methods, Evidence & Impact Faculty of Health Sciences McMaster University Hamilton, ON Canada; 3 Professor Emeritus Faculty of Health Sciences McMaster University Hamilton, ON Canada; 4 School of Rehabilitation Therapy Faculty of Health Sciences Queen's University Kingston, ON Canada; 5 Cummings School of Medicine University of Calgary Calgary, AB Canada

**Keywords:** rehabilitation, physiotherapy, occupational therapy, self-management, function, web-based application, usability, user-centered design

## Abstract

**Background:**

The global rise in the incidence of chronic conditions and aging is associated with increased disability. Physiotherapists and occupational therapists can mitigate the resulting burden on the health care system with their expertise in optimizing function. Rehabilitation self-management strategies can assist people with chronic conditions to accept, adjust, and manage different aspects of their daily functioning. Interventions delivered using technology have the potential to increase the accessibility, availability, and affordability of rehabilitation self-management support and services.

**Objective:**

This study aims to describe the development and usability evaluation of iamable, a web-based app created to provide rehabilitation self-management support for people with chronic conditions.

**Methods:**

The development and evaluation of iamable were undertaken in several phases. We used user-centered design principles and an iterative process that included consultations with rehabilitation experts; developed a prototype; and conducted usability tests, heuristic evaluations, and a focus group analysis.

**Results:**

The iamable app was developed to provide rehabilitation self-management strategies in the areas of exercise, fall prevention, fatigue management, pain management, physical activity, and stress management. We engaged adults aged ≥45 years with at least one chronic condition (N=11) in usability testing. They identified navigation and the understanding of instructions as the primary issues for end users. During the heuristic evaluation, clinicians (N=6) recommended that some areas of app content should be more succinct and that help should be more readily available. The focus group provided input to help guide clinical simulation testing, including strategies for selecting patients and overcoming barriers to implementation.

**Conclusions:**

We engaged end users and clinicians in the development and evaluation of the iamable app in an effort to create a web-based tool that was useful to therapists and their patients. By addressing usability issues, we were able to ensure that patients had access to rehabilitation strategies that could be used to help them better manage their health. Our app also provides therapists with a platform that they can trust to empower their patients to be more active in the management of chronic conditions. This paper provides a resource that can be used by others to develop and evaluate web-based health apps.

## Introduction

### Background

The global rise in the incidence of noncommunicable chronic diseases will cause an associated rise in the prevalence of disability and will be responsible for 75% of all deaths by 2030, thereby creating the most significant public health problem of the 21st century [1,2]. There is an opportunity to manage this current health care crisis through the introduction of multisector rehabilitation strategies. Physiotherapists and occupational therapists are rehabilitation professionals with expertise in promoting physical function and preventing disability, particularly in the presence of comorbid health conditions. Rehabilitation assists people with chronic conditions to accept, adjust, and manage different aspects of functioning and share similar processes that are advocated in self-management. It has been suggested that by incorporating self-management support, rehabilitation providers could provide a more effective approach to rehabilitation and chronic disease management [3-5].

Self-management is defined as an individual’s ability to manage the symptoms, treatment, physical and psychological consequences, and lifestyle changes inherent in living with a chronic condition [6]. It is usually undertaken collaboratively with the support of a health care provider. Rehabilitation principles can be incorporated into self-management support for people with chronic conditions to optimize their physical function. Most chronic conditions and disabilities are long-standing, and it is often challenging to keep people with chronic conditions engaged in long-term self-management [7]. Technology could be one way to promote ongoing engagement with rehabilitation clinicians who support self-management because it has the potential to increase the accessibility and availability of rehabilitation services. Web-based apps can ensure that patients have remote access to rehabilitative care when in-person interactions are not possible. In addition, leveraging technology may facilitate more effective self-management of chronic diseases, which may lead to improved health outcomes [8,9]. A recent systematic review that examined the effectiveness of mobile self-management apps in the long-term management of chronic conditions showed that 6 of the 9 apps developed for diabetes, chronic lung disease, and cardiovascular disease demonstrated a statistically significant improvement in the primary measure of clinical outcomes [10].

To foster the effectiveness of digital health apps, it is important to ensure their usability. A recent scoping review [11] describes the current methods used in the usability testing of eHealth apps. The methods used included questionnaires (n=105), task completion (n=57), think aloud (n=45), interviews (n=37), heuristic testing (n=18), and focus groups (n=13). Most of the studies used 1 (n=45) or 2 (n=46) testing methods. Automated mechanisms such as eye tracking were not reported as a method of assessment to test usability, and the System Usability Scale (SUS) was the most frequently used questionnaire (n=44). Multimodal usability evaluation was used most frequently, used in 67.2% (88/131) of the studies where intended users were patients and/or caregivers. The authors conclude that there is only a small proportion of reported evaluations of digital health apps in peer-reviewed publications, although the number of apps has increased substantially [9]. Further research is needed to determine the best testing methods for the usability of eHealth apps, considering their target users and their health conditions [11].

### Objective

This paper describes the development and usability evaluation of iamable, an app that uses rehabilitation strategies to promote self-management of chronic conditions. Specifically, we describe the iterative process of developing the app and conducting usability and heuristic testing. The purpose of this work is to determine whether users (people with chronic conditions) can navigate through a web-based app and access information to facilitate rehabilitation self-management. This project received ethics approval from the Hamilton Integrated Research Ethics Board (Project number: 5160).

## Methods

### Overview

**Figure 1:**
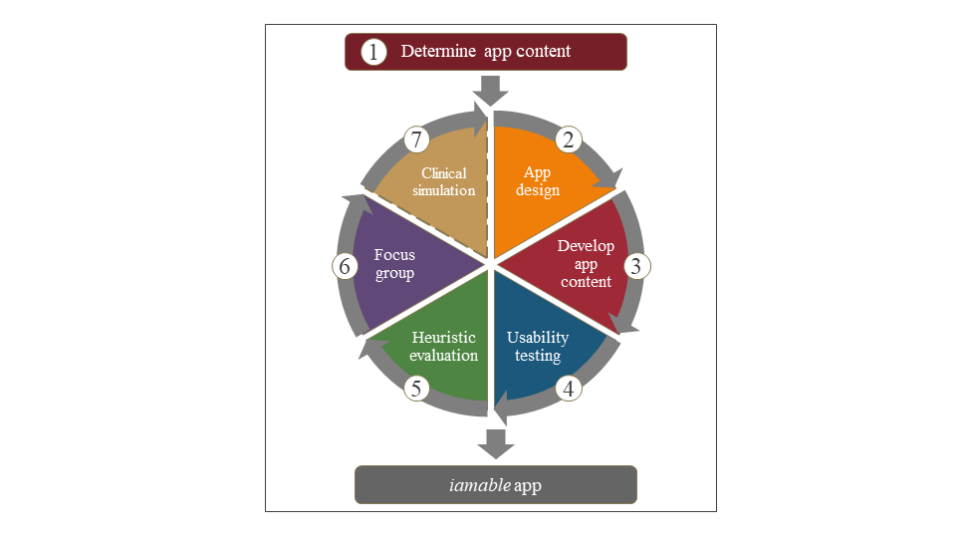
Process of iamable app development and evaluation.

The iamable app was developed and evaluated in stages, with the findings from each stage used to refine the final product. Our phased approach used user-centered design principles similar to those of Peute et al [12]. We adopted methods from the Website Developmental Model for the Health Care Consumer framework, which included (1) an information needs analysis and mock-up design, (2) website prototype development, and (3) heuristic evaluation and think-aloud analysis. This phased approach was developed further. It is illustrated as an iterative process in Figure 1. This paper reports on stages 1 to 6.

### Determining App Content

We undertook a consultation process with physiotherapists and occupational therapistswho were either clinical or research experts in the self-management of chronic conditions to prioritize the topics for module development that formed the content for the app. In creating the self-management modules, we used the concept of clinical concordance to develop rehabilitation strategies used by either physiotherapists or occupational therapistsor both professionals to address issues faced by people with chronic conditions (who frequently have comorbid conditions). Clinical concordance is an approach that uses the concepts of clinical discordance (where conditions are not linked by pathogenesis or management) and concordance (conditions represent an overall pathophysiological risk profile) [13]. The unique contributions of physiotherapists and occupational therapiststo self-management interventions target mobility, functional activity, and participation levels [5]. Often, the rehabilitation strategies suggested by physiotherapists and occupational therapistshave a high level of therapeutic concordance in that they can be applied (sometimes with some modification) to multiple conditions where there may be clinical discordance, but the functional outcomes are similar.

Briefly, the consultation was a web-enabled consensus process, whereby specific self-management strategies that could be used to manage mobility, functional activity, and participation issues associated with chronic conditions were identified by the participants. The 6 self-management strategies identified were physical activity, fall prevention, fatigue management, pain management, stress management, and exercise. The concept behind self-management is that people with chronic conditions all experience to a greater or lesser extent some homogenous symptoms, including fatigue, pain, and stress; therefore, the app was designed to provide people with rehabilitation-focused strategies they could use to break the symptom cycle [14].

### App Design

**Figure 2:**
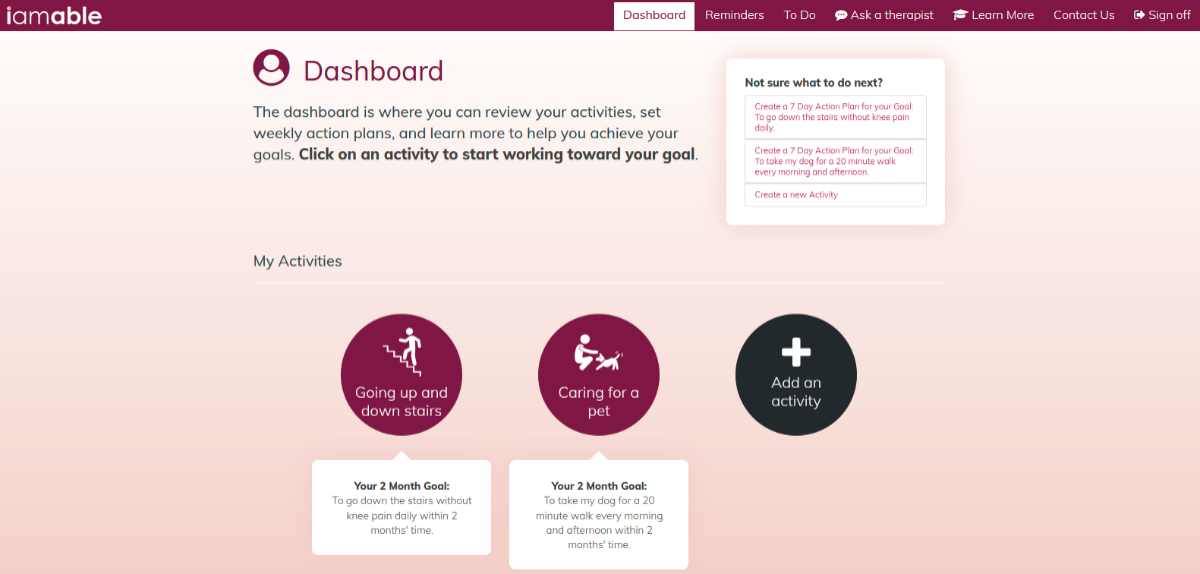
Sample screenshot of the iamable user interface.

We began by identifying the goals of the project with the design team from Media Production Services at McMaster University. Our primary objective was to develop a web-based app that would benefit both patients and clinicians. We wanted to provide patients with access to a trusted source of health information and rehabilitation strategies that they could use to optimize their function and participation in daily life. We also wanted patients to have a tool that would help them self-monitor their health and communicate with their therapist to better manage their chronic condition. In addition, we wanted to create a resource that busy rehabilitation clinicians could use to support the self-management efforts of their patients. With the design team, we identified the target users as patients receiving rehabilitation services in primary care and discussed web design features that would appeal to this population. We worked together to create wireframes (illustrations of basic page layouts) and detailed mock-ups to visualize the user experience and the information architecture of the app. We used the Drupal Content Management System [15], which allowed us flexibility in our design. The app was built to be mobile and responsive, with a focus on tablet and computer use. We focused on maximizing white space with minimalistic design and colors to limit confusion and provide obvious focus points on the page and chose a simple san-serif font face and larger font sizes where possible with increased contrast to support target audiences from an older demographic. We then worked with the design team to develop a prototype and engaged 2 end users and 2 clinicians to review the mock-ups and provide feedback. They were able to comment on the general appearance of the user interface (UI) and provide input regarding basic navigation (moving between screens) and workflow processes (completing forms). Figure 2 shows a sample screenshot of the finalized design for the iamable UI (Multimedia Appendix 1 provides additional screenshots of the UI).

### Developing App Content

The content of the iamable app was developed using concepts from social cognitive theory, including goal setting and self-monitoring [16]. Users were prompted to identify an activity that was important to them that they were having difficulty performing because of their health problem and to create a long-term goal for that activity. They were asked to identify gaps in their knowledge and explore the information contained in the self-management modules. The development of the modules (pain management, exercising with a chronic condition, physical activity and chronic conditions, stress management, fatigue management, and fall prevention) was undertaken by rehabilitation experts in their respective fields, who used systematic reviews and clinical practice guidelines to convey strategies and recommendations to help users manage their condition(s). Each evidence-informed module was developed in the same format so that users of the app would (1) complete self-assessments to receive feedback about their level of risk for health outcomes and receive advice tailored to their needs, (2) have access to evidence-based self-management strategies, (3) engage in action planning based on activities with which they self-identified, and (4) have the option to consult with a physiotherapist or occupational therapist via secure messaging. All content was reviewed by the research team before it was finalized and before the usability evaluation.

### Usability Testing

Usability testing was conducted with adults aged ≥45 years (N=11) recruited from a database of previous research participants who consented to future contact and through an advertisement distributed to the network of the Hamilton Council on Aging. All potential participants were screened by telephone to determine if they met the following inclusion criteria: (1) the presence of at least one chronic condition and (2) computer proficiency. To be eligible to participate, people were required to respond “somewhat easily” or “very easily” to all questions on the Computer Proficiency Questionnaire-12 (CPQ-12) [17]. Participants were also asked to estimate their daily technology use (Multimedia Appendix 2 [17] provides a copy of CPQ-12).

Usability testing was performed in the Advanced Human-Computer Interaction (HCI) Lab, located in the DeGroote School of Business on McMaster University’s main campus in Hamilton, Ontario. The HCI Lab consisted of a participant room and a control room, with one-way mirrors between the rooms to allow for the monitoring of subjects and their interactions. Audiovisual recordings of the test sessions were obtained from 4 video cameras at multiple angles and a recording of the website screen overlaid with eye tracking (Tobii Eye-tracker, Tobii AB; Multimedia Appendix 3 provides a detailed usability test plan).

We used the concurrent think-aloud method, which is one of the most frequently used tests of usability in health care research [18]. Participants were required to verbalize their actions and thoughts, as they completed representative tasks using the app. Users were asked to perform the following tasks: (1) sign in to the app; (2) complete step 1: select 1 activity that you are having difficulty with because of your health problem (The app allows users to select up to 3 activities in step 1. We asked test users to select 1 activity for the usability testing session); (3) step 2: rate your ability to perform the activity on a 10-point scale and set a goal; (4) select the activity goal that you would like to work toward; (5) select one of the self-management modules you identified that would help you reach your goal; (6) complete self-assessment; (7) on the basis of the results of self-assessment, select a topic such as “medications” in the Fall Prevention module to learn more about medications and fall risk; (8) create a 7-day action plan; and (9) ask your therapist a question (send your therapist a message). Participants completed a training task to practice the think-aloud method [19] before the testing session began, and the interviewer provided feedback on the participant’s performance. Participants were prompted to continue talking if they were silent for more than 10-15 seconds. Each testing session took approximately 1.5 hours.

Participants completed the SUS at the conclusion of the test session. The SUS is a tool that quickly and easily evaluates a user’s subjective rating of a product’s usability [20]. It consists of 10 statements that are scored on a 5-point Likert scale (1=strongly disagree and 5=strongly agree). Estimates of reliability range from 0.83 to 0.97 [21]. Scores for the SUS can range from 0 to 100, where higher scores indicate better usability (Multimedia Appendix 4 [20] provides an adapted copy of the SUS).

Audio recordings of the usability test sessions were transcribed to create log files, which were then marked up by a research coordinator who reviewed the video recordings and added annotations about usability issues identified by the user. The transcripts were coded to identify specific types of usability issues, as proposed by Kushniruk et al [22] (Multimedia Appendix 5 [22] provides details of the video coding scheme). Qualitative comments were extracted from the log files during the coding procedures if they were illustrative and representative of the usability issue.

### Heuristic Evaluation

The next phase of the usability assessment involved a heuristic evaluation, which is a usability inspection method used to identify usability problems in the UI design. Evaluators examined the interface and assessed the degree to which it complied with established usability principles (the heuristics) [23]. A total of 8 heuristics for evaluation focused on identifying specific usability problems, and 4 heuristics focused on identifying issues related to health literacy*.* The criteria were adapted from the work of Nielsen et al [18,23-25]. We recruited clinicians with experience using digital health apps and rehabilitation self-management interventions (N=6) to perform an independent evaluation of the iamable app based on these 12 criteria. The clinicians included a convenience sample of 3 physiotherapists and 3 occupational therapists(female: n=5; male: n=1; clinical experience: mean 14 years; range 3-31 years) who worked in primary care and/or whose practice involved the management of people with chronic conditions who might use the app. The clinicians practiced in both urban and rural primary care settings, and several clinicians provided care to marginalized populations.

Clinicians were asked to log in to the iamable app remotely and complete six tasks: (1) identify an activity; (2) rate the activity and set a goal; (3) navigate to the self-management module, complete the self-assessment, and review the 2 module topics assigned; and (4) create an action plan. Each clinician evaluated 2 of the self-management modules, so that each module was evaluated by 1 physiotherapist and 1 occupational therapist. They systematically progressed through the screens required to complete the task. When they encountered a usability issue, they recorded it on the heuristic evaluation form, applied a severity rating (1=mild, 2=moderate, and 3=severe), captured a screenshot of the page on which they encountered the problem, and provided comments or recommendations to resolve the issue (Multimedia Appendix 6 provides a copy of the heuristic evaluation form). We allowed approximately 1.5 hours for completion of these tasks.

Clinicians submitted their completed evaluation forms electronically. Heuristic violations were summed and tabulated according to severity, and clinicians’ recommendations were summarized by the research team.

### Focus Group

We held a focus group using videoconferencing 1 week after the completion of the heuristic evaluation to gather the clinicians’ general impressions of the app and its clinical utility. Specifically, the questions they were asked were as follows: What are the strengths of the app? What are the areas that could be improved? Which patients do you think might benefit from using the app? Which patients would you recommend it to? How do you envisage using the app in your clinical practice?

## Results

### Usability Testing

Table 1 presents the demographic characteristics of the participants. The mean age of the participants was 69.3 years (SD 10.1 years; range 48-84 years), and the majority were retired (8/11, 73%) and used technology for 1-5 hours daily. The chronic conditions they reported included arthritis (7/11, 64%), hypertension (5/11, 45%), diabetes (3/11, 27%), asthma (3/11, 27%), chronic obstructive pulmonary disease (2/11, 18%), heart disease (1/11, 9%), and other (5/11, 45%; included chronic neck or back pain, chronic kidney disease, anxiety, and depression).

Table 2 provides an example of task duration. Users were asked to complete the self-assessment, and duration was measured as the length of time it took users to read the instructions and click the self-assessment button on the module page. The duration varied considerably between users, ranging from 21 to 264 seconds (average task duration for females: 79.8 seconds; average task duration for males: 108 seconds), indicating that users follow instructions and navigate the app with differing levels of efficiency.

**Table 1:** Participant characteristics (N=11).

Characteristic		Values
Age (years), mean (SD)		69.3 (10.1)
**Sex, n (%)**		
	Male	6 (55)
	Female	5 (45)
**Marital status, n (%)**		
	Married	8 (73)
	Divorced	2 (18)
	Widowed	1 (9)
**Employment, n (%)**		
	Working part-time	2 (18)
	Retired	8 (73)
	On disability	1 (9)
**Ethnicity, n (%)**		
	European	11 (100)
**Financial situation, n (%)**		
	Have just enough to get along	3 (27)
	Are comfortable	8 (73)
**Number of chronic diseases, n (%)**		
	1	3 (27)
	2	3 (27)
	3	3 (27)
	4	2 (18)
**Daily technology use (hours), n (%)**		
	<1	1 (9)
	1-3	4 (36)
	3-5	4 (36)
	>5	2 (18)

**Table 2:** Example of task duration.

User	Age (year)	Sex	Number of chronic conditions	Task duration (seconds)
1	84	Female	4	48
4	77	Female	1	141
6	70	Female	4	42
10	56	Female	1	33
11	65	Female	3	135
2	72	Male	2	171
3	77	Male	2	34
5	72	Male	2	68
7	73	Male	1	264
8	48	Male	3	90
9	72	Male	3	21

**Figure 3:**
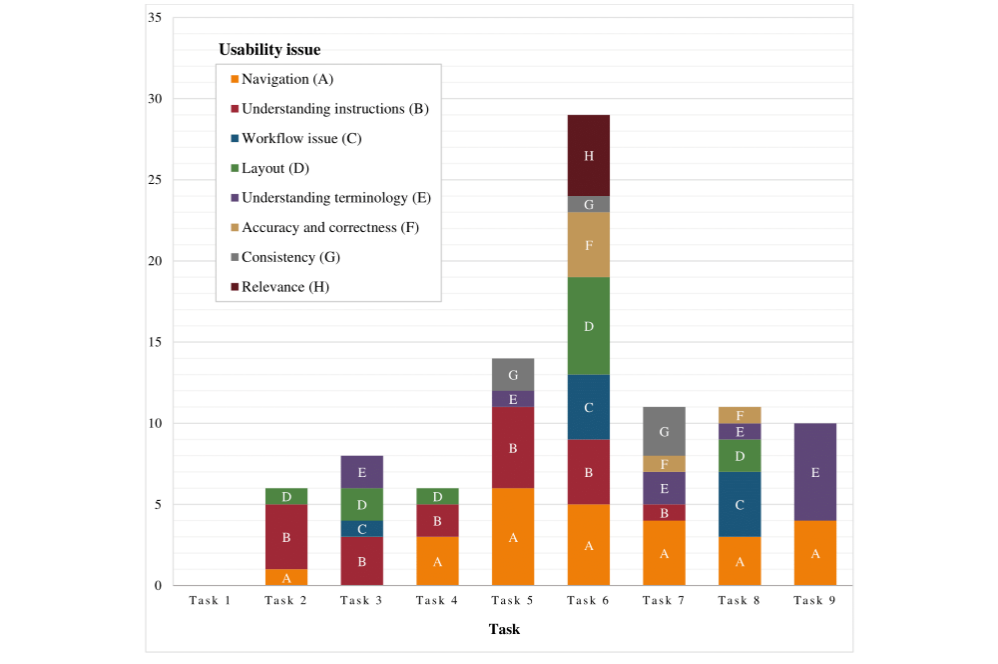
Summary of usability issues by task. Task 1: log in; task 2: select activity; task 3: rate activity and set goal; task 4: open activity: task 5: select self-management module; task 6: complete self-assessment; task 7: select module topic; task 8: create action plan; task 9: message therapist.

In total, 7 usability issues emerged from the think-aloud transcripts. They include navigation, understanding instructions, layout, understanding terminology, workflow issues, accuracy and correctness, and consistency. The usability issues for each of the 9 tasks are illustrated in Figure 3. The most common usability issue across tasks was related to navigation. Users described difficulty in determining what to do next, uncertainty about which content was clickable, and confusion about the labels on certain buttons and tabs. The task with the greatest number of usability issues was task 6: complete self-assessment. The most common usability issue when asked to complete the self-assessment was related to layout, with some users unable to locate the self-assessment button on the page, whereas others had difficulty finding their self-assessment results. Other usability issues with this task included difficulties with navigation, understanding instructions, workflow, and accuracy. Examples of usability issues by task were extracted from the audio recordings of the think-aloud sessions and are presented in Tables 3 and 4.

**Table 3:** Think-aloud results for tasks 2-6.

Task	Usability issue	Description	Example
Task 2: Select activity	Understanding instructions	Users had difficulty understanding that they should select an activity that they were having difficulty with because of their health.	“Like gardening, I’m not that thrilled about gardening and I’m not very good at it. Would that be considered one, or no?” (User 7) “It’s asking for an activity you are having difficulty doing because of your health.” (RC^a^) “Oh, I’m sorry. I didn’t even think of that. Ok, because of my health.” (User 7)
Task 3: Rate activity and set goal	Understanding instructions	Users had difficulty determining how many modules they should select.	“So, you want me to pick just one?” (User 5) “No, you can pick as many as you would like.” (RC)
Task 4: Open activity	Navigation	Users did not realize that they needed to click on the icon to open the activity.	“So, we’ve done that...I don’t see a ‘next’ to click on, so what should we do about that? How do we move on?” (User 1)
Task 5: Select self-management module	Navigation	Users had difficulty locating the self-management modules on the page.	“That’s this, right? That’s what this is?” (User 3) “So, that’s to create an action plan…” (RC) “Oh, over here. Oh, ok. So that’s the module.” (User 3)
Task 5: Select self-management module	Understanding instructions	Users had difficulty with the instructions because they had the option to explore the topics or complete the self-assessment.	“So I’m not really sure here.” (User 5) “So based on the information you read...” (RC) “Complete the self-assessment, it says. So I guess they want me to do this, right?” (User 5)
Task 6: Complete self-assessment	Navigation	Users suggested that the recommendation provided by the system after completing the self-assessment did not provide adequate direction to help navigate to the next task.	“So, should I go back to self-assessment? This is the same page as before, right? I don’t understand where I go from here.” (User 7)
Task 6: Complete self-assessment	Understanding instructions	Users had difficulty following the instruction to complete the self-assessment. Some were drawn to the “Not sure what to do next” box and the instruction to create an action plan instead.	“Ok, ‘Start by completing the self-assessment. Based on your answers, you will receive a recommendation to guide you in selecting the topics below that will help you the most.’ Ok, so this is what I’ve got to do, create a 7 Day Action Plan for your goal.” (User 11)
Task 6: Complete self-assessment	Layout	Users had difficulty finding the self-assessment button.	“But if the instruction says ‘Start by completing the self-assessment’, can you see on the page where that might be?” (RC) “Right here. So [the button] is not in a place that I would have thought to look. It’s in a place where I think to not pay attention.” (User 11)
Task 6: Complete self-assessment	Workflow	Users suggested that questions on the self-assessment were unclear.	“This is a little confusing...there could be an easier way to get the answers.” (User 7)
Task 6: Complete self-assessment	Accuracy	Users identified that one of the self-assessments was not scoring correctly, providing some users with the wrong result.	“Yeah, that’s kind of worrying when I read that...I’m thinking, how can I be sedentary when I’m doing something 7 days a week.” (User 2)

^a^RC: research coordinator.

**Table 4:** Think-aloud results for tasks 7-9.

Task	Usability issue	Description	Example
Task 7: Select module topic	Navigation	Users were unsure how to navigate to topics (this task required the user to click on a topic to open it).	“Ok, so I don’t understand what I’m doing next...because am I going to open one of these by clicking?” (User 11)
Task 7: Select module topic	Consistency	Users expressed difficulty because the topic buttons did not appear to be hyperlinks.	“Ok, well the first thing is...is that a link? When you look at that, you think that it is just text...so that changes the way that I was thinking.” (User 2)
Task 8: Create action plan	Navigation	Users were confused by the delete action plan button, not sure what to do after creating an action plan.	“I don’t want to delete this. So where else do I want to go? I can either delete it, or I create a 7-Day Action Plan which I already did. I don’t know where to go now. Unless you want to delete this goal.” (User 7)
Task 7: Select module topic	Workflow issue	Users anticipated a menu of action plans to choose from rather than having to create one of their own.	“So, would this answer not have...would I not have been led to this, from maybe, from the results of the previous step? Would I not have been given some suggested actions at this point?” (User 9)
Task 9: Message therapist	Navigation	Users were not certain that the ask button should be used to contact the therapist.	“Ask...that means ask a therapist? Ok...so you type the question and she’ll get back to you online?” (User 1)
Task 9: Message therapist	Understanding terminology	Users were confused about the save and send buttons when attempting to send a message to the therapist.	“So I’m saving it rather than sending it...But I will click send because that’s the only thing I can think of to get it off to the therapist.” (User 11)

We were not able to fully test the messaging function in the app, as the test user accounts were not linked to therapist accounts. In clinical practice, the user will be able to post a question to the therapist, who will, in turn, receive notification about the message and enter the app to provide a response. Users will be reminded that they can expect to receive a response from their therapist within 2 working days; if they have an urgent issue that requires an immediate response, they will be told to contact their therapist directly.

The SUS provided a single score that estimated the overall usability of the iamable app. The mean score of the iamable app (N=11) was 71.14 (SD 19.67). Products that are acceptable have SUS scores above 70, with superior products scoring in the high 70s to the high 80s [26].

### Heuristic Evaluation

**Figure 4:**
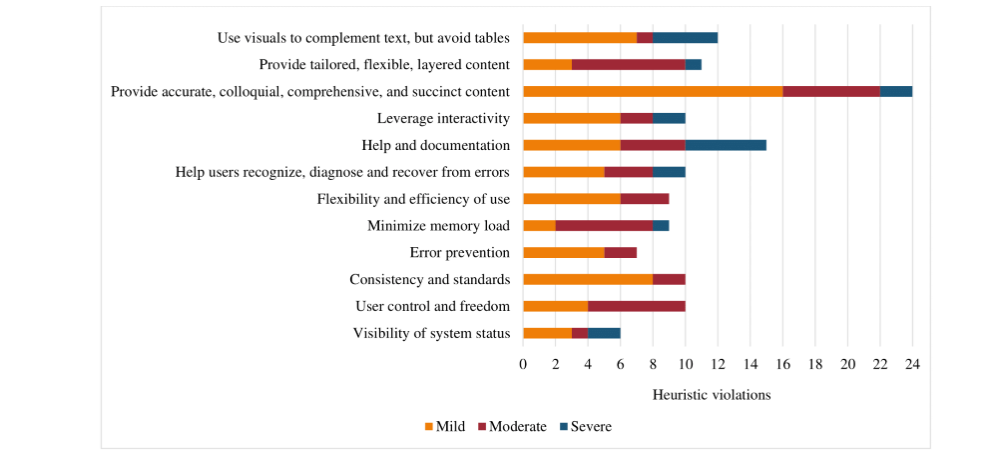
Results of heuristic testing. The graph depicts the overall frequency of heuristic violations.

Figure 4 shows the results of the heuristic evaluation performed by the 6 clinicians. Specifically, it shows the overall frequency of heuristic violations identified by clinicians for all tasks that they were required to complete. Violations were rated according to severity: 1=mild, 2=moderate, and 3=severe. The heuristic that resulted in the highest number of violations (n=24) across all 4 tasks was “provide accurate, colloquial, comprehensive, succinct content.” The heuristic that resulted in the fewest violations (N=6) was the “visibility of system status,” where the user is informed as to the state of the system at any given moment (Multimedia Appendix 7 provides a frequency table summarizing all heuristic violations).

**Figure 5:**
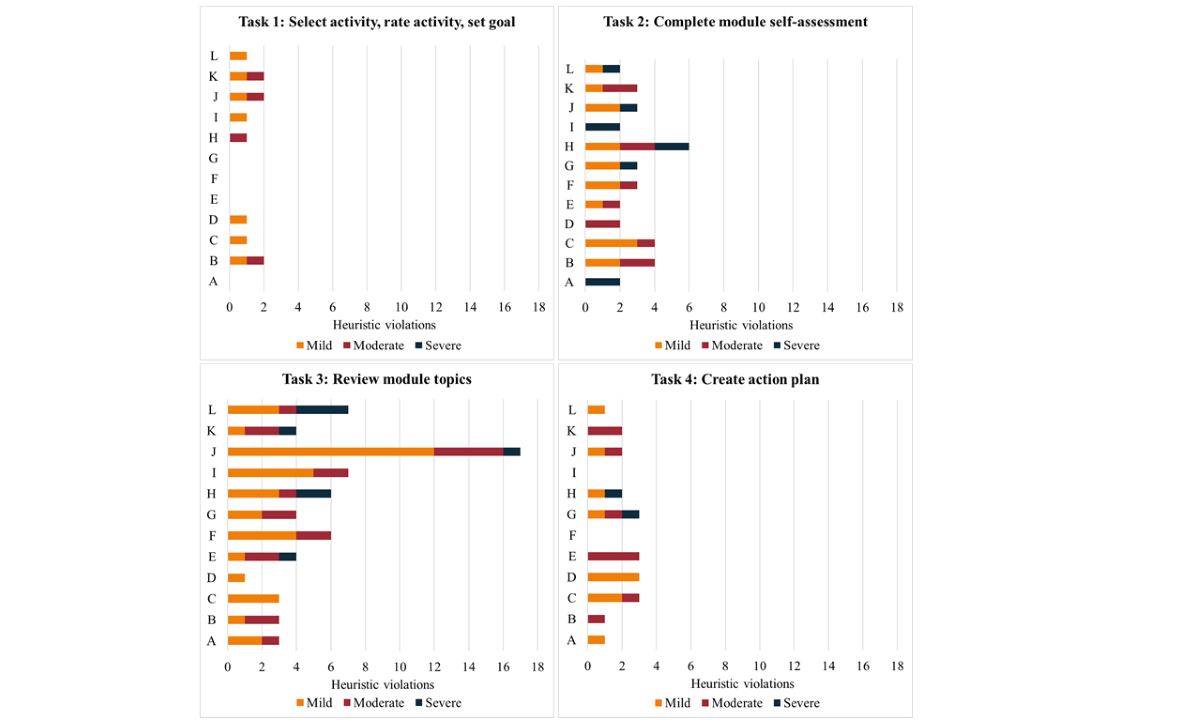
Heuristic violations by task. "A" refers to the visibility of system status. "B" refers to user control and freedom. "C" refers to consistency and standards. "D" refers to error prevention. "E" refers to minimized memory loads. "F" refers to the flexibility and efficiency of use. "G" refers to helping users recognize, diagnose, and recover from errors. "H" refers to help and documentation. "I" refers to leveraging interactivity. "J" refers to providing accurate, colloquial, comprehensive, and succinct content. "K" refers to providing tailored, flexible, and layered content. "L" refers to using visuals to complement text but avoiding tables.

The raters identified 36 heuristic violations for task 2 (complete module self-assessment) with mostly mild to moderate severity ratings (Figure 5). Violations for the heuristic “help and documentation” indicated that there was no help button or frequently asked question (FAQ) option, and the raters felt that a video or timer should be added to facilitate the self-assessment task (in the fall prevention module). The raters identified severe violations for the heuristic “visibility of system status” because the instructions to complete the self-assessment remained on the screen and the self-assessment button remained active even after the self-assessment was completed. The other severe violation related to the heuristic “leverage interactivity,” where the clinician felt the recommendation following the self-assessment was not sufficiently tailored (exercise module; Multimedia Appendix 8 provides additional details).

Task 3 (review module topics) generated the greatest number of heuristic violations (n=65) from the raters, the majority of which were mild (Figure 5). The heuristic “provide accurate, colloquial, comprehensive and succinct content” was identified as a mild violation by all raters for each of the modules they reviewed. Feedback from raters included the need for a more lay-friendly language to avoid using language that labels users (ie, faller vs nonfaller), to provide examples or links to explain more difficult concepts (ie, cognition and corticosteroids), to shorten videos and text in some areas, and to provide emphasis to ensure that users do not take certain actions (ie, stop taking a medication) without first consulting with their physician. Raters identified several severe violations, including (1) the heuristic “use visuals to complement text” where a trip hazard was not identified in a home hazards video (fall prevention module) and the video was not uploaded (stress management module), (2) the heuristic “minimize memory load” because there is no way for users to track their progress through the module (ie, an indicator that a topic had been read or completed), and (3) the heuristic “help and documentation” because there is no topic-specific help or FAQs (Multimedia Appendix 8 provides additional details).

Raters detected 21 mild or moderate heuristic violations for task 4 (creating an action plan; Figure 5). Raters noted severe violations in relation to the heuristic “help and documentation” (n=1) because there was no help option available and the heuristic “system provides a clear and easy to understand way of recovering from an error” (n=1) after the system did not save the action plan after prompting that the confidence level was too low (Multimedia Appendix 8 provides additional details).

### Focus Group

#### Strengths of the App

Clinicians identified several positive features of the app. They reported that the videos were particularly helpful, especially because the content was simplified and summarized for the user. For example, they stated that the self-management modules explained concepts using simple, nontechnical language, and when it was not possible to replace technical terms, a simple definition was provided in brackets.

They appreciated the interactive nature of the app, which enabled the user to set goals, receive tailored information, and check their progress. An example they gave of the tailoring was if a user scored high on the pain catastrophizing scale (the pain self-assessment), they were directed to complete the topics focusing on thoughts, emotions, and pain. They endorsed the self-assessments associated with each module and noted that the information from these would be helpful to both the user and the clinician to have an assessment of baseline functioning and track progress toward goals.

#### Areas for Improvement

Although the clinicians were very positive about the videos, they commented that some were too long, estimating that the maximum time that a video would sustain a user’s attention would be 2 minutes. They suggested that it would be helpful if there was a way for users to mark their progress within the app, so that if they returned, they would know where to resume (ie, by providing a bookmark, by changing the color of the font, or by identifying favorite content). They suggested that more consistent use of the “add to my reminders” feature would help users identify and prioritize tasks that they needed to undertake to help them complete their action plans and reach their goals. They felt that the app might increase engagement in exercise and self-management and adherence in the management of their chronic condition.

Some therapists felt the recommendation following the self-assessment in the exercise module provided the same information irrespective of the score and that it would be helpful if the exercise prescription was better tailored to the user’s fitness level.

Several clinicians noted technical issues when using the app on their phone or tablet, where certain buttons did not function, and it was difficult to read the content without having to manually resize the text.

#### Suitability for Patient Groups

Primary care was recommended as the setting in which the app would be the most useful. Clinicians agreed that they would want to introduce the app to patients in a 1:1 consultation to ensure that patients had the ability to use it successfully before engaging with it independently. They reported that there were no contraindications for any patient group, although the level of digital and health literacy required to use the app might prove too advanced for some of the populations seen in primary care, such as new immigrants or people who were homeless. Clinicians thought that an app such as iamable might be especially appealing to users who had social anxiety. They commented that users in rural and remote areas would find the app very helpful because they could use it in a clinical setting and then have it as support to work on their goals from home. Clinicians agreed that the app would help keep patients engaged in their rehabilitation between visits. Finally, the clinicians suggested translating the content of the app to other languages.

## Discussion

### Principal Findings

This study describes the successful development of an app to support the self-management of people with chronic conditions and address symptoms that could be managed using rehabilitation strategies. The approach we used to evaluate the usability of the iamable app included task completion, think-aloud, interviews, heuristic testing, and focus groups, a process similar to that of Peute and endorsed by Maramba [11,12]. iamable is the first app developed and systematically evaluated by rehabilitation professionals that addresses the holistic management of people with chronic conditions using rehabilitation strategies. The 6 areas of self-management targeted by the app align with the self-management skills outlined by Lorig for breaking the symptom cycle [14]. The premise of the relationship between these symptoms is that, irrespective of the disease, people with chronic conditions often experience common issues. The modules included within the app are evidence based, and the strategies recommended to address these issues have been developed using best practice guidelines (ie, prescribing multicomponent home-based exercise and promoting home safety assessments to reduce the risk of falls [27] and providing pain neuroscience education to help patients manage chronic pain [28]). The app requires users to identify the gaps in their knowledge and select modules accordingly. The design of the iamable app was iterative and involved consultation with users from the early stages of development, with revisions and changes based on their feedback and input. Our multistage approach to usability evaluation enabled us to make modifications serially and remediate user problems before the next stage of testing.

### Common Issues Identified by Usability Testing

The two most frequent issues encountered by users during usability testing were navigation and the understanding of instructions within the app. These issues are well documented in the literature and may result from reduced vision, hearing loss, or psychomotor impairment [29]. Barriers related to navigation and instructions on websites and health apps have been reported in other older adult populations, suggesting the need for special considerations and adaptations to improve these and other fundamental aspects of app design [30,31]. Our results are not surprising because older people in the United States and Europe report that they are less likely to engage with web-based apps because of the perceived complexity of internet use [32,33]. Older people are less likely than younger people to perceive websites as user friendly [34]. In an effort to improve the usability of the iamable app, we addressed issues with navigation and understanding instructions that were identified during usability testing. Modifications made to improve navigation included making buttons clearly clickable; using more consistent use of hyperlink colors; modifying button size, color, and location to draw the user’s attention; and relabeling buttons to clarify their meaning. Color and size sensitivity reduce with age, especially blue-green differentiation [29]. To clarify the instructions, we added text such as “select all that apply” and “start by completing the self-assessment” to improve usability. These changes will further provide accommodations to older users with diminished abilities in attention, learning, and memory, which manifest in determining where to go next [29].

A study by Bangor et al [26] found that there was a small, significant correlation between age and SUS scores (SUS scores decreased with increasing age) but no effect of gender. In our sample, we observed a sex difference where women had higher SUS scores compared with men (women: mean 78.5, SD 17.6; men: mean 65.0, SD 20.7) despite having similar levels of comorbidity and education and slightly older mean age (women: mean 70.4 years, SD 10.8 years; men: mean 68.3 years, SD 10.4 years). A mean SUS score of 71.14 (SD 19.67) suggested that the usability of the iamable app is acceptable. An SUS score of 71.4 (SD 11.6) corresponds with a rating of “good” using an adjective rating scale [26]. The SUS was administered to participants at one point in time only; therefore, it is reasonable to assume that with the revisions made to the app following usability testing, SUS scores would likely have improved if the survey had been readministered.

For the sample task in which we calculated task duration (ie, complete the self-assessment), there were no differences by age or number of chronic conditions. In addition, when the outlier was removed, there were no sex differences in task completion time.

### Most Common Heuristic Violations

The top heuristic violations identified by the clinicians were related to the provision of accurate, colloquial, comprehensive, succinct content and help and documentation. The heuristic “provide accurate, colloquial, comprehensive, succinct content” targets health literacy and is intended to ensure that written information is brief, relevant, and in the users’ vernacular. Our goal was to engage users in the app content in a way that motivates them to create goals and complete action plans. To do this, we endeavored to create content that was both brief and to the point, actionable, and engaging [12]. It is also suitable for users with a range of literacy skills to reflect the types of patients that therapists would see in clinical practice. In response to clinician feedback, the iamable app content was revised to add images to complement text, make the text more concise, simplify instructions, and emphasize safety considerations. In addition, the heuristic “help and documentation” directly impacts the usability and is intended to ensure that help is available to users when needed*.* In response to comments from clinicians, we clarified for users that they should use the “Ask a Therapist” feature for self-management support and added a “Contact Us” page so that users could access technical assistance when required.

### Focus Group

The focus group with the clinicians provided us with additional information about the app and recommendations for further changes and development that we would not have gathered through heuristic testing alone. Clinicians agreed that some patients would be able to use the app independently. A systematic review reported that self-management apps have been used successfully both with and without clinician input [10], but older people are more likely to engage in learning and adopt the use of technology if they live with someone or have assistance [35]. In response to concerns about ease of navigation, we are exploring ways to make it easier for users to find where they left off during previous sessions using the app. The focus group also provided us with information to help guide the final stage of the iamable evaluation, the clinical simulation. Participants were able to provide advice about overcoming barriers to implementation from the perspective of both the clinician and the patient. Potential barriers for clinicians included increased workload to support patients using the app and the need to obtain permission from clinic administrators. Patient barriers might include cost, literacy, language, connectivity, and availability or accessibility issues, which are similar to other reports in the literature [10].

### Limitations

There were some limitations to this study. The sample recruited for usability testing was not ethnically diverse. All participants were of European heritage and were English speaking. To ensure that the iamable app is accessible to a diverse range of users, a future study that includes a more heterogeneous sample is warranted. Furthermore, we were unable to use eye tracking data to substantiate our results. For reasons related to cost and analyst availability, we were not able to analyze the eye tracking data that we collected. Eye tracking provides an automated method for objectively evaluating usability. Eye tracking analysis would have resulted in a more comprehensive usability evaluation of the iamable app. Combining eye tracking with qualitative data can provide a more holistic understanding of usability issues. For example, Cho et al [36] used eye tracking metrics (time to first fixation, time spent, revisits, and total number of fixations) to compare groups with and without task difficulty to help identify barriers to task performance.

For usability testing, we required that our sample have a high level of computer proficiency, which likely meant that they had access to technology. Overall, 73% (8/11) of our sample reported that they used their computers and mobile devices on average for 1-5 hours per day. With future testing, it will be important to include older people with less access to technology to ensure usability for those with low digital literacy. Finally, we made many revisions to the app in response to the results of usability testing and feedback from the heuristic evaluation but did not repeat either assessment to determine whether these changes resolved the identified issues.

The final stage of our usability evaluation will be a clinical simulation where we will enlist therapist-patient dyads (n=26) from primary care sites where occupational therapists and physiotherapists engage in rehabilitation self-management. As was recommended in the focus group, the plan will be to have therapists introduce the iamable app in a face-to-face or virtual session with the patient and then to provide support as needed over the course of the trial. This will enable us to determine more specifically the role the app might play in a primary care environment, how the therapists will use the app with patients who have different chronic conditions, and how useful the app is for patients who are currently receiving rehabilitation. This final stage of the usability evaluation is considered important by the study team because the app has not yet been tested as an interactive therapeutic resource where the therapist and patient engage collaboratively in chronic disease self-management.

### Conclusions

In conclusion, we have produced an app that closely matches the expectations of both the patient and clinician end users, and although there are usability issues identified from our testing, the app was rated highly on the validity of its evidence-based content. The information contained in the app can provide self-management support to people with chronic conditions with or without the support of a therapist because some users may not be receiving care from a rehabilitation practitioner. The app has the potential to both reinforce information provided during a rehabilitation intervention and provide rehabilitation strategies to users who can engage in self-management independently. It may strengthen the patient’s relationship with the therapist, increase engagement, and enable the patient to proactively address concerns related to their rehabilitation [37]. A systematic review reported that self-management apps have been used successfully both with and without clinician input. Although clinicians noted that they would introduce the app to their patients, some patients would be able to use it independently without prior instruction [10]. Adoption of the iamable app may face barriers to use such as cost, literacy, language, connectivity, availability and accessibility issues, similar to other health apps reported in the literature [10].

To guide the development of the iamable app, we incorporated user-centered design principles [12]. We used an iterative process that included consultation with rehabilitation experts to determine app content, app design, usability testing, and heuristic evaluation plus a focus group. This paper provides a resource that can be used by others to develop and evaluate web-based health apps that can benefit both patients and clinicians.

## Appendix

Multimedia Appendix 1 Screenshots of the iamable user interface.

Multimedia Appendix 2 Computer Proficiency Questionnaire.

Multimedia Appendix 3 Usability test plan.

Multimedia Appendix 4 System Usability Scale.

Multimedia Appendix 5 Video coding scheme.

Multimedia Appendix 6 Heuristic evaluation form.

Multimedia Appendix 7 Frequency of heuristic violations.

Multimedia Appendix 8 Examples of heuristic violations by task.

## Abbreviations

AGE-WELL: Aging Gracefully Across Environments Using Technology to Support Wellness, Engagement and Long Life
CPQ-12: Computer Proficiency Questionnaire-12
FAQ: frequently asked question
HCI: Human-Computer Interaction
MIRA: McMaster Institute for Research on Aging
SUS: System Usability Scale
UI: user interface
