# Supplementary material for: Using a Web-Based App to Deliver Rehabilitation Strategies to Persons With Chronic Conditions: Development and Usability Study
Source: JMIR Rehabil Assist Technol. 2021 Mar 18;8(1):e19519. doi: 10.2196/19519 (PMC8294797; doi:10.2196/19519)

Multimedia Appendix 1: Screenshots of *iamable* user interface

www.iamable.ca

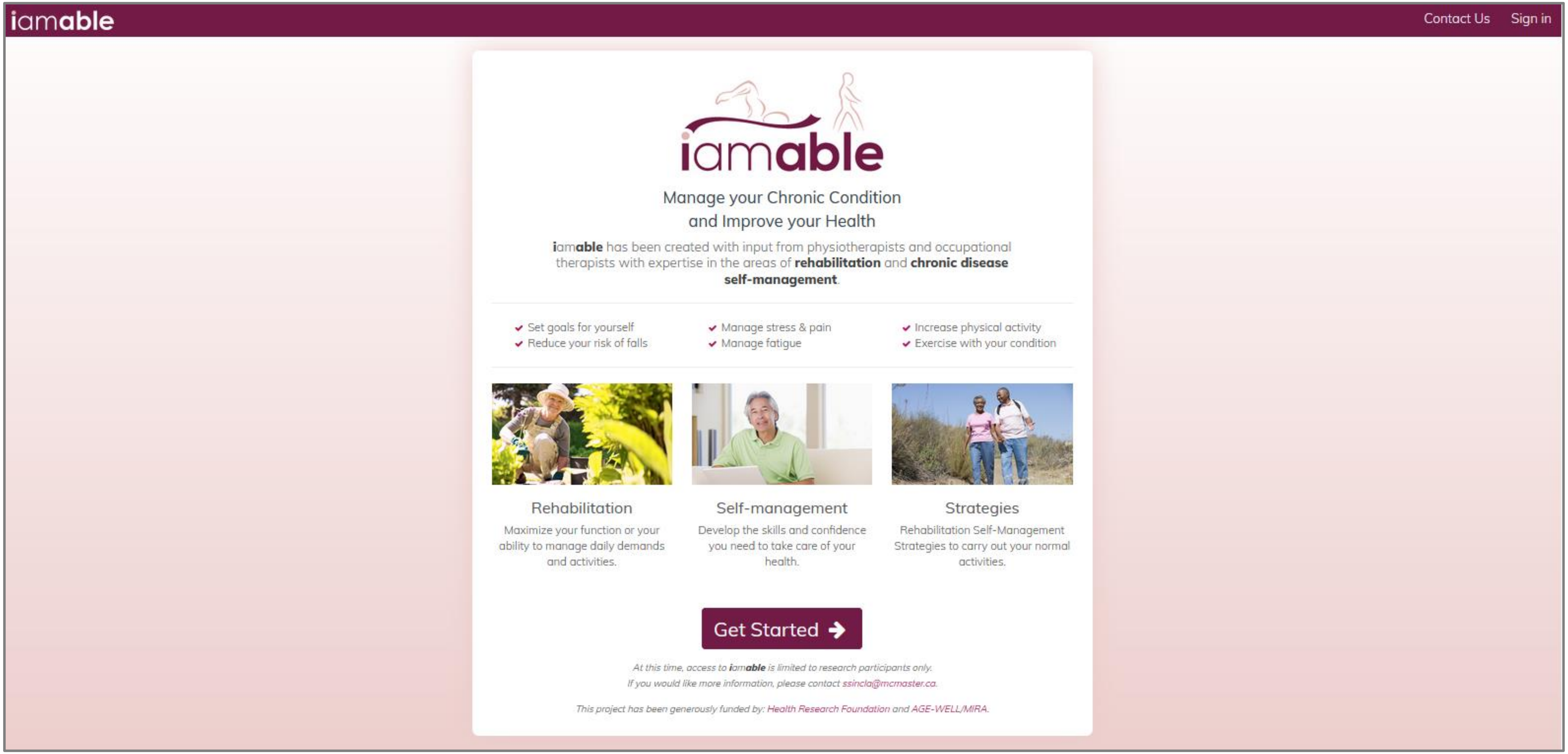

Dashboard

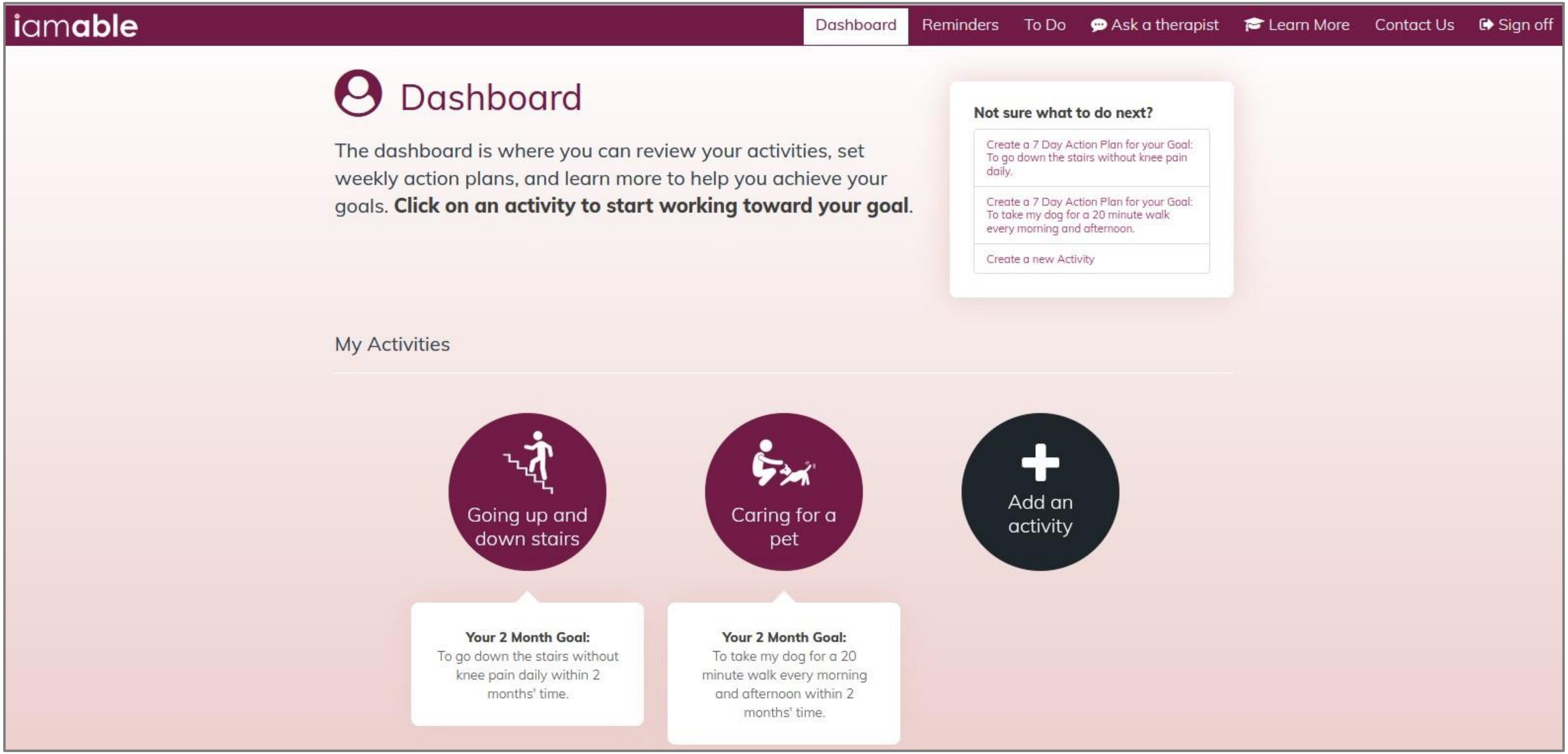
iamable
Dashboard
Reminders
To Do
Ask a therapist
Learn More
Contact Us
Sign off
Dashboard
The dashboard is where you can review your activities, set weekly action plans, and learn more to help you achieve your goals. Click on an activity to start working toward your goal.
Not sure what to do next?
Create a 7 Day Action Plan for your Goal: To go down the stairs without knee pain daily.
Create a 7 Day Action Plan for your Goal: To take my dog for a 20 minute walk every morning and afternoon.
Create a new Activity
My Activities
Going up and down stairs
Caring for a pet
Add an activity
Your 2 Month Goal:
To go down the stairs without knee pain daily within 2 months' time.
Your 2 Month Goal:
To take my dog for a 20 minute walk every morning and afternoon within 2 months' time.

Main activity page

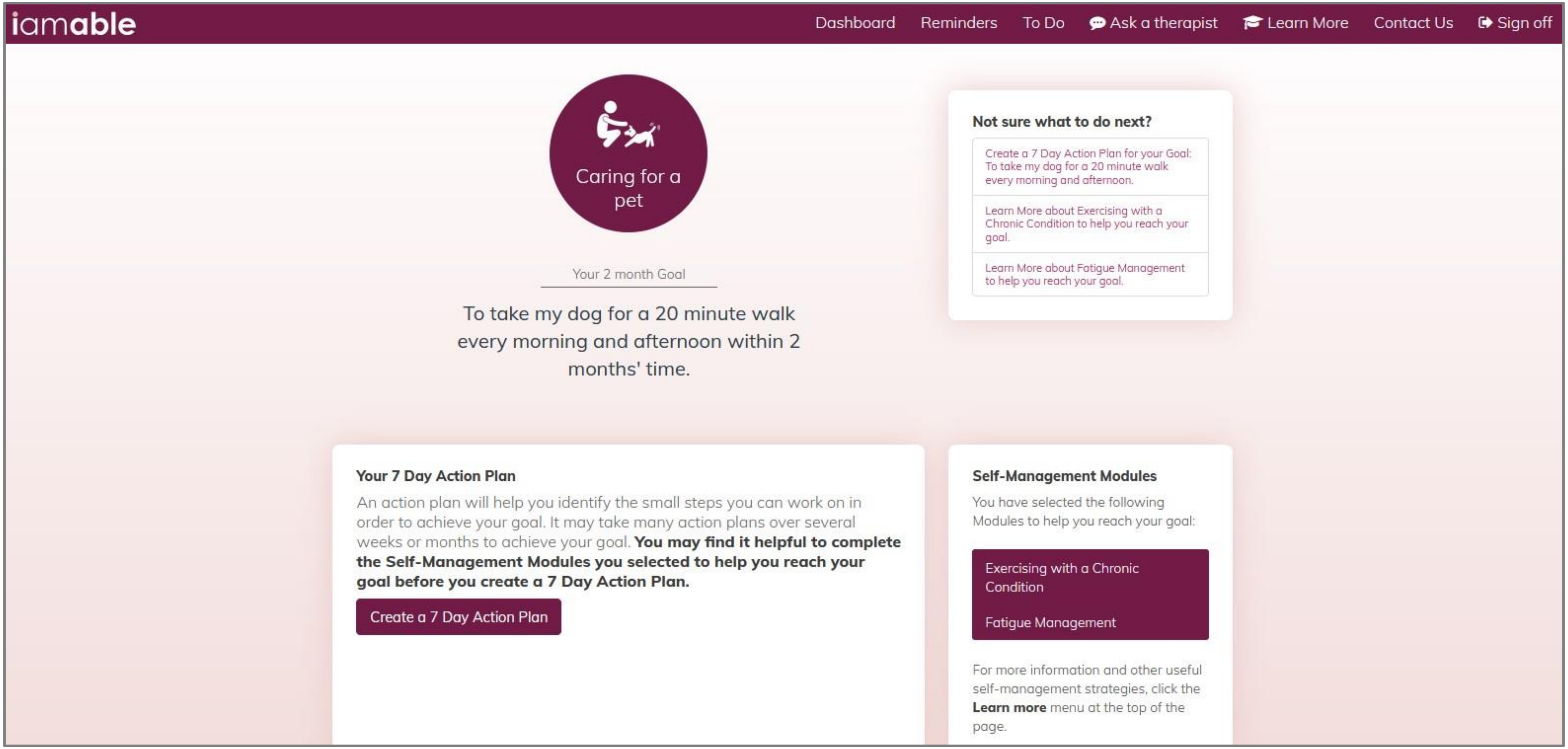

Exercising with a Chronic Condition module

iamable

Dashboard | Reminders | To Do | Ask a therapist | Learn More | Contact Us | Sign off

Exercising with a Chronic Condition

Exercise falls into four basic categories—**cardiovascular**, **strength**, **flexibility** and **balance**. Most people tend to focus on one type of exercise and think they're doing enough. Each type is different, though, and doing them all will give you more benefits.

What type of exercise will help you reach your goal? Click on the type of exercise to get more information.

To learn more about balance exercises, to go to the Fall Prevention Module.

Exercise Topics

1. The difference between physical activity and exercise
2. The benefits of exercise
3. Is it safe for me to exercise?
4. Understanding your exercise prescription
5. Warming up & cooling down
6. Managing symptoms during exercise
7. Overcoming barriers to exercise

Types of Exercise

- Upper Body Flexibility
- Upper Body Strength
- Lower Body Flexibility
- Lower Body Strength
- Cardiovascular Fitness

Fatigue Management module

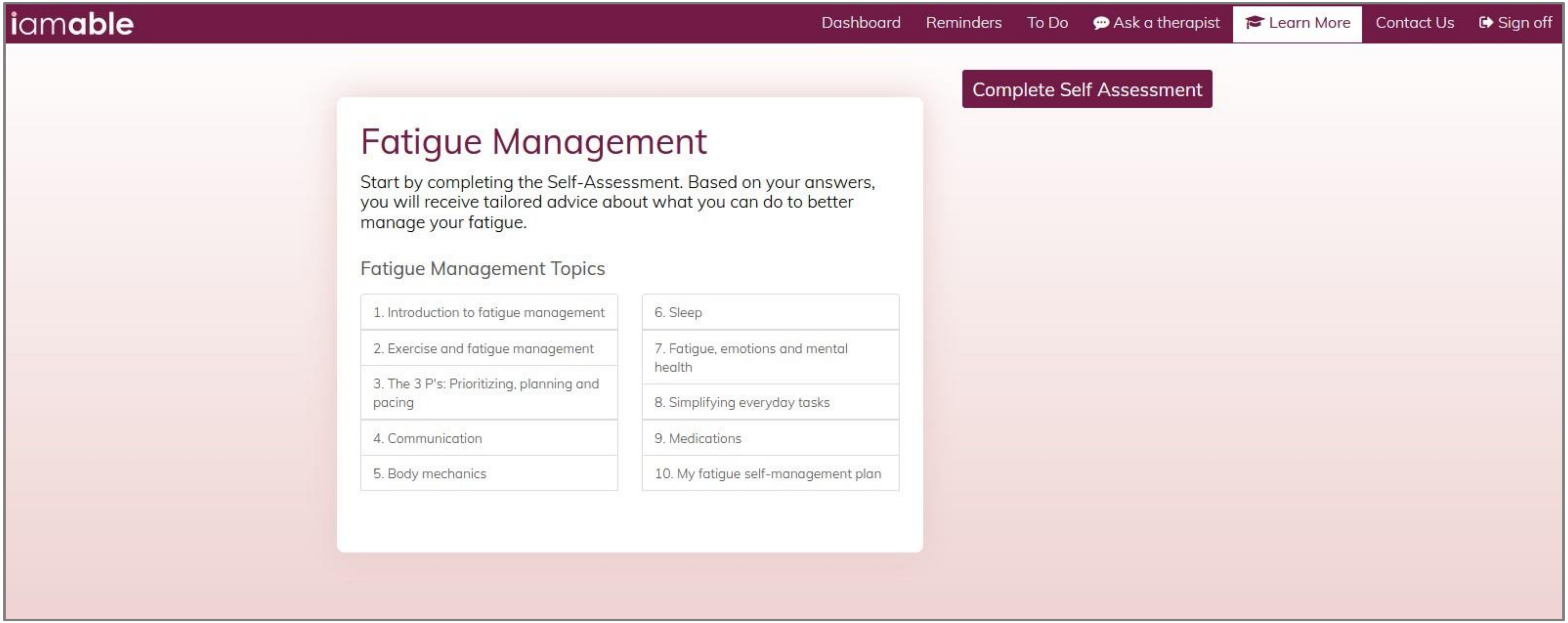

Supplement: Multimedia Appendix 1 [file rehab_v8i1e19519_app1.pdf]
