# Supplementary material for: Using a Web-Based App to Deliver Rehabilitation Strategies to Persons With Chronic Conditions: Development and Usability Study
Source: JMIR Rehabil Assist Technol. 2021 Mar 18;8(1):e19519. doi: 10.2196/19519 (PMC8294797; doi:10.2196/19519)
Supplement: Multimedia Appendix 2 [file rehab_v8i1e19519_app2.docx]

**Appendix 2:** Computer/Tablet Proficiency Questionnaire (CPQ-12)

| How easily can you perform these tasks with your computer or tablet? | Never tried | Not at all | Not very easily | Somewhat easily | Very easily |
| --- | --- | --- | --- | --- | --- |
| Computer/tablet basics | | | | | |
| Use a keyboard to type | 1 | 2 | 3 | 4 | 5 |
| Use a mouse (if applicable) | 1 | 2 | 3 | 4 | 5 |
| Communication | | | | | |
| Open emails | 1 | 2 | 3 | 4 | 5 |
| Send emails | 1 | 2 | 3 | 4 | 5 |
| Internet | | | | | |
| Find information about community resources on the Internet | 1 | 2 | 3 | 4 | 5 |
| Find information about my health on the Internet | 1 | 2 | 3 | 4 | 5 |
| Entertainment | | | | | |
| Use a computer/tablet to watch movies and videos | 1 | 2 | 3 | 4 | 5 |
| Use a computer/tablet to listen to music | 1 | 2 | 3 | 4 | 5 |
| Printer | | | | | |
| Load ink into the printer | 1 | 2 | 3 | 4 | 5 |
| Fix the printer when paper jams | 1 | 2 | 3 | 4 | 5 |
| Calendar | | | | | |
| Use a computer/tablet to enter events into a calendar | 1 | 2 | 3 | 4 | 5 |
| Check the date and time of upcoming and prior appointments | 1 | 2 | 3 | 4 | 5 |

(Boot et al, 2015)
