# Supplementary material for: Using a Web-Based App to Deliver Rehabilitation Strategies to Persons With Chronic Conditions: Development and Usability Study
Source: JMIR Rehabil Assist Technol. 2021 Mar 18;8(1):e19519. doi: 10.2196/19519 (PMC8294797; doi:10.2196/19519)
Supplement: Multimedia Appendix 3 [file rehab_v8i1e19519_app3.docx]

**Appendix 3:** Usability Test Plan

**Scope:**

Web Application: <http://www.iamable.ca>

**Purpose:** To determine if users can navigate through the application and access information that can be used to facilitate chronic disease self-management.

1. Can users log-in to the application?
2. Can users independently identify an activity and set a goal?
3. Can users navigate to the self-management modules?
4. Do users choose to complete the self-assessment?
5. Can users access information about the module topic?
6. Can users create an action plan?

**Session Length:** 60-90 minutes per user

- Consent process
- Instructions to participant
- Training task (complete a task on the McMaster Athletics and Recreation website to practice the Think Aloud method)
- Usability test
- Complete System Usability Scale

**Equipment:**

- Desktop computer (Monitor: 24” TFT widescreen; Resolution: 1080p (1920 x 1200 pixels); Browser: Internet Explorer Version 11; OS: Windows 8.1; Software: Tobii Pro Studio (eye-tracking and screen recording) (Tobii AB, Stockholm, Sweden).
- Video recording from 4 angles

**Participants:**

- 8-10 users
- Adults and older adults with a chronic condition
- All items on Computer Proficiency Questionnaire (Boot et al, 2015) scored as *Somewhat Easily* or *Very Easily*.

**Tasks:**

1. Sign in to the application. (provide sign-in credentials)
2. Complete Step 1: Select **1** activity that you are having difficulty with because of your health problem.
3. Complete Step 2: Rate the activity and set a goal. (only prompt if unable to proceed)
4. Select the goal that you would like to work toward (only prompt if unable to proceed)
5. Select **1** of the Self-Management Modules you identified that would help you reach your goal.
6. Complete the Self-Assessment (only prompt if unable to proceed)
7. Based on results of Self-Assessment, select a topic to learn more about (only prompt if unable to proceed)
8. Create a 7-Day Action Plan.
9. Ask your therapist a question (send your therapist a message).

**Outcomes:**

Metrics:

- Successful task completion
- Time on task
- System Usability Scale (Brooke, 1996)
