# Supplementary material for: Using a Web-Based App to Deliver Rehabilitation Strategies to Persons With Chronic Conditions: Development and Usability Study
Source: JMIR Rehabil Assist Technol. 2021 Mar 18;8(1):e19519. doi: 10.2196/19519 (PMC8294797; doi:10.2196/19519)
Supplement: Multimedia Appendix 4 [file rehab_v8i1e19519_app4.docx]

**Appendix 4:** System Usability Scale

Please circle the number that reflects your immediate response to each statement.

- Don’t think too long about each statement.
- Make sure you respond to every statement.
- If you don’t know how to respond, simply circle number “3.”

|  | Strongly  disagree | |  | | | Strongly  agree | |
| --- | --- | --- | --- | --- | --- | --- | --- |
| 1. I think that I would like to use this app (*iamable*) frequently | 1 | 2 | | 3 | 4 | | 5 |
| 1. I found the app unnecessarily complex | 1 | 2 | | 3 | 4 | | 5 |
| 1. I thought the app was easy to use | 1 | 2 | | 3 | 4 | | 5 |
| 1. I think that I would need the support of a technical person to be able to use this app | 1 | 2 | | 3 | 4 | | 5 |
| 1. I found the various functions in this app were well integrated | 1 | 2 | | 3 | 4 | | 5 |
| 1. I thought there was too much inconsistency in this app | 1 | 2 | | 3 | 4 | | 5 |
| 1. I would imagine that most people would learn to use this app very quickly | 1 | 2 | | 3 | 4 | | 5 |
| 1. I found the app very awkward to use | 1 | 2 | | 3 | 4 | | 5 |
| 1. I felt very confident using the app | 1 | 2 | | 3 | 4 | | 5 |
| 1. I needed to learn a lot of things before I could get going with this app | 1 | 2 | | 3 | 4 | | 5 |

(Adapted from Brooke, 1996)
