# Supplementary material for: Using a Web-Based App to Deliver Rehabilitation Strategies to Persons With Chronic Conditions: Development and Usability Study
Source: JMIR Rehabil Assist Technol. 2021 Mar 18;8(1):e19519. doi: 10.2196/19519 (PMC8294797; doi:10.2196/19519)
Supplement: Multimedia Appendix 5 [file rehab_v8i1e19519_app5.docx]

**Appendix 5:** Video Coding Scheme

| Usability Issues | | Examples |
| --- | --- | --- |
| N | **NAVIGATION** – the user has problems moving through a system or user interface. | Difficulty finding buttons or content on screen |
| CON | **CONSISTENCY** – the user has problems due to a lack of consistency in the user interface. | User expected text to be hyperlinked |
| TERM | **MEANING OF ICONS/TERMINOLOGY** – the user does not understand language/labels used in the interface. | Meaning of save/send in messages; add to my reminders in module topics |
| EM | **UNDERSTANDING ERROR MESSAGES** – the user does not understand meaning of error messages. | User does not see error message. |
| UI | **UNDERSTANDING INSTRUCTIONS** – the user does not understand user instructions. |  |
| WI | **WORKFLOW ISSUES** – there are issues with system workflow negatively impacting user interaction. | Difficulty completing self-assessment or setting goal, creating action plan |
| G | **GRAPHICS** – there are issues with graphics. |  |
| L | **LAYOUT** – there are problems with the layout of screens or information on those screens. | Helper text, confusion re: sample action plans, results of self-assessment not obvious, required to scroll to read |
| RT | **SPEED/RESPONSE TIME** – the system is slow or response time is an issue. |  |
| COL | **COLOR** – the user does not like color or color schemes used in the interface. |  |
| F | **FONT** – the font is too small or not readable. |  |
| Usefulness of Content | | |
| A | **APPLICABILITY** – information presented is not applicable to user’s situation |  |
| AC | **ACCURACY/CORRECTNESS** – information or advice provided by system is not correct or accurate. | Results of self-assessment incorrect |
| R | **RELEVANCE** – information presented by a system is not relevant to their carrying out their task. | Results of self-assessment don’t provide adequate direction re: next step |
| TIME | **TIMELINESS** – information is not timely. |  |
| I | **IMPACT ON WORK ACTIVITIES** –unexpected impact of the system on task completion. |  |
| Error Codes | | |
| S | **SLIP** – the user has made a mistake but corrects the mistake. |  |
| M | **MISTAKE** – the user has made a mistake that is not corrected. |  |
| W | **WORKAROUND** – the user is not using the approach to carrying out task that is recommended by the system. | Navigating to complete task in a way other than expected |

(Kushniruk et al, 2015)
