# Supplementary material for: Using a Web-Based App to Deliver Rehabilitation Strategies to Persons With Chronic Conditions: Development and Usability Study
Source: JMIR Rehabil Assist Technol. 2021 Mar 18;8(1):e19519. doi: 10.2196/19519 (PMC8294797; doi:10.2196/19519)
Supplement: Multimedia Appendix 7 [file rehab_v8i1e19519_app7.docx]

**Appendix 7:** Frequency of Heuristic Violations

| Heuristic | Task 1 | | | Task 2 | | | Task 3 | | | Task 4 | | |
| --- | --- | --- | --- | --- | --- | --- | --- | --- | --- | --- | --- | --- |
| Severity | 1 | 2 | 3 | 1 | 2 | 3 | 1 | 2 | 3 | 1 | 2 | 3 |
| 1. Visibility of system status: The user is informed as to the state of the system at any given moment. |  |  |  |  |  | 2 | 2 | 1 |  | 1 |  |  |
| 1. User control and freedom: The user should feel in control of the system. | 1 | 1 |  | 2 | 2 |  | 1 | 2 |  |  | 1 |  |
| 1. Consistency and standards: The user interface and basic system operations should be consistent. | 1 |  |  | 3 | 1 |  | 3 |  |  | 2 | 1 |  |
| 1. Error prevention: The interface is designed to prevent errors from occurring. | 1 |  |  |  | 2 |  | 1 |  |  | 3 |  |  |
| 1. Minimize memory load: The user interface supports recognition rather than recall. |  |  |  | 1 | 1 |  | 1 | 2 | 1 |  | 3 |  |
| 1. Flexibility and efficiency of use: The user interface should be customizable and flexible for different types of users. |  |  |  | 2 | 1 |  | 4 | 2 |  |  |  |  |
| 1. Help users recognize, diagnose and recover from errors. |  |  |  | 2 |  | 1 | 2 | 2 |  | 1 | 1 | 1 |
| 1. Help and documentation: Help should be available to users when needed. |  | 1 |  | 2 | 2 | 2 | 3 | 1 | 2 | 1 |  | 1 |
| 1. Leverage interactivity. | 1 |  |  | 0 |  | 2 | 5 | 2 |  |  |  |  |
| 1. Provide accurate, colloquial, comprehensive, succinct content. | 1 | 1 |  | 2 |  | 1 | 12 | 4 | 1 | 1 | 1 |  |
| 1. Provide tailored, flexible, layered content. | 1 | 1 |  | 1 | 2 |  | 1 | 2 | 1 |  | 2 |  |
| 1. Use visuals to complement text, but avoid tables. | 1 |  |  | 1 | 0 | 1 | 3 | 1 | 3 | 1 |  |  |

Task 1: Select activity, rate activity, set goal; Task 2: Complete module self-assessment; Task 3: Review module topics; Task 4: Create an action plan. Severity: 1 = Mild, 2 = Moderate, 3 = Severe.
