# Supplementary material for: Using a Web-Based App to Deliver Rehabilitation Strategies to Persons With Chronic Conditions: Development and Usability Study
Source: JMIR Rehabil Assist Technol. 2021 Mar 18;8(1):e19519. doi: 10.2196/19519 (PMC8294797; doi:10.2196/19519)
Supplement: Multimedia Appendix 8 [file rehab_v8i1e19519_app8.docx]

**Appendix 8:** Examples of Heuristic Violations by Task

**Task 1:** Select activity, rate activity, set goal

| Heuristic | Example with severity rating |
| --- | --- |
| User control and freedom | Unable to edit activity once selected (Moderate); No back function, required to use browser’s back button (Mild) |
| Consistency and standards | Responsive design not evident when using mobile devices (Mild) |
| Help and documentation | No help button or FAQ option (Moderate) |
| Provide accurate, colloquial, comprehensive, succinct content. | Language used in the outcome measure (PSFS) might be confusing to users (Moderate); Users might have difficulty verbalizing their goals (Mild) |
| Provide tailored, flexible, layered content | Content available in English only (Moderate) |

**Task 2:** Complete module self-assessment

| Heuristic | Example with severity rating |
| --- | --- |
| Visibility of system status | Direction to complete self-assessment (SA) appears on screen even after SA completed (Severe) |
| User control and freedom | No back function, required to use browser’s back button (Mild); No option to save progress without submitting form (Mild) |
| Consistency and standards | Complete Self-Assessment button difficult to find on the page (Moderate); Self-assessment results difficult to find on the page (Mild) |
| Help users recognize, diagnose and recover from errors | No option to edit or delete self-assessment once submitted (Mild) |
| Help and documentation | No help button or FAQ option (Severe); Add video link or timer to facilitate self-assessment task (Mild) |
| Leverage interactivity | Recommendation following self-assessment not sufficiently tailored (Exercise module) (Severe) |
| Provide tailored, flexible, layered content | Content wasn’t as personalized as expected (Moderate); Content available in English only (Moderate) |
| Provide accurate, colloquial, comprehensive, succinct content | Ensure that self-assessment instructions are easy to understand; emphasize safety (Severe); Module may not be accessible for some clients (Pain module) (Severe) |
| Use visuals to complement text, but avoid tables | Pictures in the self-assessments would be useful (Mild) |

**Task 3:** Review module topics

| Heuristic | Example with severity rating |
| --- | --- |
| Visibility of system status | No way of monitoring progress through topics (i.e. change of colour, tick box) (Mild) |
| User control and freedom | Instructions that include “go to…” should include hyperlink to that page (Mild); Inconsistent use of “Add to My Reminders” function (Moderate) |
| Consistency and standards | Inconsistent use of bold or colored font for hyperlinks (Moderate); content moves down the page after self-assessment has been completed (Mild) |
| Minimize memory load | No instruction re: what to do next upon completion of module (Moderate); Add feature that helps indicate which topics have been completed (Severe) |
| Flexibility and efficiency of use | No option to increase size of Topics pop-up window (Mild); consider option to identify “favourite” content (Moderate) |
| Help users recognize, diagnose and recover from errors | Hyperlink disabled (Severe) |
| Help and documentation | No help button or FAQ option (Severe) |
| Leverage interactivity | Worksheets are not fillable/cannot be saved on the system (Mild) |
| Provide accurate, colloquial, comprehensive, succinct content | Language should be more lay-friendly (Mild); Avoid use of language that labels users (i.e. faller vs. non-faller) (Mild); Some content too long (videos, text) (Moderate); Some images do not depict ideal posture (Moderate); Ensure users understand they should not stop taking medications prior to discussion with GP (Severe) |
| Provide tailored, flexible, layered content | Exercise programs should be more tailored (Severe); Content available in English only (Moderate) |
| Use visuals to complement text, but avoid tables | Questioning value of infographic (Exercise module) (Mild); Concern that the kitchen stool in the home hazards video was not identified as a possible trip hazard (Severe); Video not uploaded (Stress Mgmt. module) (Severe) |

**Task 4:** Create an action plan

| Heuristic | Example with severity rating |
| --- | --- |
| Visibility of system status | No indication that action plan log has been saved (Mild) |
| User control and freedom | No option to edit or delete action plan (Moderate) |
| Consistency and standards | Instruction at the end of the action plan video is not correct (Moderate) |
| Error prevention | Data can be entered in log on a future date (Mild) |
| Minimize memory load | Cannot easily return to action plan form after navigating away (Moderate); confidence level described in video but should also be explained on action plan form (Moderate) |
| Help users recognize, diagnose and recover from errors | System did not save action plan after prompting that confidence level was too low (Severe); System allowed creation of action plan that was nonsensical (Moderate) |
| Help and documentation | No help button or FAQ option (Moderate) |
| Provide accurate, colloquial, comprehensive, succinct content | Users may not understand that their 7-day action plan is for the next 7 days (Moderate) |
| Provide tailored, flexible, layered content | Content available in English only (Moderate) |
| Use visuals to complement text, but avoid tables | Newly created action plan not completely obvious on the page (Mild) |
